# Beneficial Effects of Resveratrol Administration—Focus on Potential Biochemical Mechanisms in Cardiovascular Conditions

**DOI:** 10.3390/nu10111813

**Published:** 2018-11-21

**Authors:** Michał Wiciński, Maciej Socha, Maciej Walczak, Eryk Wódkiewicz, Bartosz Malinowski, Sebastian Rewerski, Karol Górski, Katarzyna Pawlak-Osińska

**Affiliations:** 1 Department of Pharmacology and Therapeutics, Faculty of Medicine, Collegium Medicum in Bydgoszcz, Nicolaus Copernicus University, M. Curie 9, 85-090 Bydgoszcz, Poland; wicinski4@wp.pl (M.Wi.); maciej.walczak5@hotmail.com (M.Wa.); bartosz.malin@gmail.com (B.M.); srewerski@gmail.com (S.R.); karolgorski-2@gazeta.pl (K.G.); 2 Department of Obstetrics, Gynecology and Gynecological Oncology, Faculty of Medicine, Collegium Medicum in Bydgoszcz, Nicolaus Copernicus University, Ujejskiego 75, 85-168 Bydgoszcz, Poland; msocha@copernicus.gda.pl; 3 Department of Pathophysiology of Hearing and Balance System, Faculty of Medicine, Collegium Medicum in Bydgoszcz, Nicolaus Copernicus University, M. Curie 9, 85-090 Bydgoszcz, Poland; osinskak1@wp.pl

**Keywords:** resveratrol, cardiovascular, inflammation, cytokines, pathways

## Abstract

Resveratrol (RV) is a natural non-flavonoid polyphenol and phytoalexin produced by a number of plants such as peanuts, grapes, red wine and berries. Numerous in vitro studies have shown promising results of resveratrol usage as antioxidant, antiplatelet or anti-inflammatory agent. Beneficial effects of resveratrol activity probably result from its ability to purify the body from ROS (reactive oxygen species), inhibition of COX (cyclooxygenase) and activation of many anti-inflammatory pathways. Administration of the polyphenol has a potential to slow down the development of CVD (cardiovascular disease) by influencing on certain risk factors such as development of diabetes or atherosclerosis. Resveratrol induced an increase in Sirtuin-1 level, which by disrupting the TLR4/NF-κB/STAT signal cascade (toll-like receptor 4/nuclear factor κ-light-chain enhancer of activated B cells/signal transducer and activator of transcription) reduces production of cytokines in activated microglia. Resveratrol caused an attenuation of macrophage/mast cell-derived pro-inflammatory factors such as PAF (platelet-activating factor), TNF-α (tumour necrosis factor-α and histamine. Endothelial and anti-oxidative effect of resveratrol may contribute to better outcomes in stroke management. By increasing BDNF (brain-derived neurotrophic factor) serum concentration and inducing NOS-3 (nitric oxide synthase-3) activity resveratrol may have possible therapeutical effects on cognitive impairments and dementias especially in those characterized by defective cerebrovascular blood flow.

## 1. Introduction

Resveratrol (3,5,4′-trihydroxy-trans-stilbene) is a natural non-flavonoid polyphenol and phytoalexin produced by a considerable number of plants in response to stress factors such as pathogens or injury [1,2]. The substance can be found in peanuts, grapes, red wine and some berries [3]. It has been proven to be a potent antioxidant [4], antiplatelet [5,6] and anti-inflammatory agent [7] in vitro. Despite numerous studies, mechanisms of resveratrol action have not been clearly identified. According to the results of pharmacokinetic analysis, resveratrol undergoes rapid metabolism in the body, its bioavailability after oral administration is very low despite of absorption reaching 70%, which undermines the physiological significance of the high concentrations used in in vitro studies [6]. Mentioned effects are probably a result of its ability to purify the body from ROS [8,9], inhibition of COX [10,11] and activation of many anti-inflammatory pathways, including among others: SIRT-1 (Sirtuin-1) [12]. SIRT-1 disrupts the TLR4/NF-κB/STAT signal which subsequently leads to the reduction of produced cytokines in activated microglia [13], or macrophage/mast cell-derived pro-inflammatory factors such as platelet-activating factor PAF, TNF-α and histamine [14]. 

**Figure 1:**
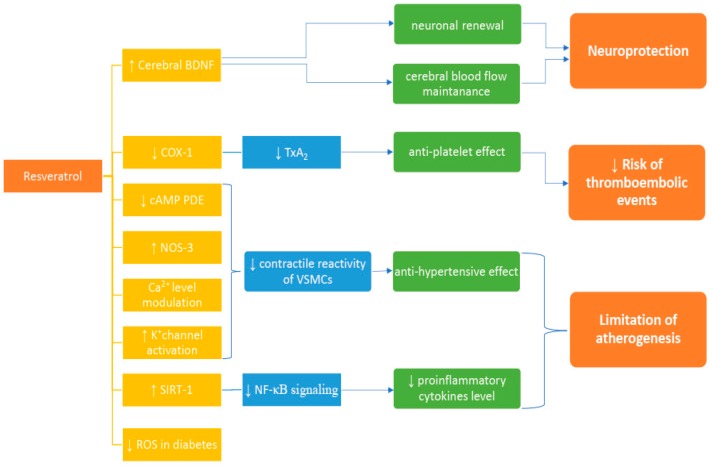
Proposed mechanisms of resveratrol activity**.** COX-1: cyclooxygenase type 1; cAMP: cyclic adenosine monophosphate; PDE: phosphodiesterase; SIRT-1: sirtuin-1; NOS-3: Nitric oxide synthase, ROS: reactive oxygen species, NF-κB: nuclear factor kappa-light-chain-enhancer of activated B cells; TxA_2_: thromboxane A_2_; VSMCs: vascular smooth muscle cells; ↓: a decrease; ↑: an increase.

Cardiovascular diseases are the most common cause of death in the world, it is estimated that about 18 million people died because of CVD in 2016. It is 31% of all deaths worldwide. Over 17 million (39%) of premature deaths (under 70 years) due to non-communicable diseases are caused by CVD [15]. Regardless of the significant improvement and great emphasis on CVD treatment, the statistics show that searching for new ways to help cardiovascular patients is essential. Resveratrol has a potential to slow down the development of CVD by influencing on certain risk factors. In this article, the authors present the potential mechanisms of resveratrol’s activity (presented in Figure 1).

## 2. Inflammation

Atherosclerosis is a multifactorial disease of the vascular walls leading to the development of plaques and consequent stenosis of the arteries [16,17]. Current progress in basic science has signified essential role of inflammation in initiation, progression and finally possible thromboembolic complications of the disease. Atherosclerosis-related inflammation is mediated by various cytokines which include among others: TNF-α, interleukin-6 (IL-6), monocyte chemoattractant protein-1 (MCP-1) as well as factors inducing the expression of intercellular adhesion molecule 1 (ICAM-1), vascular cell adhesion molecule 1 (VCAM-1) and E-selectin adhesion molecules. Long-term studies in humans conducted by Tomé Carneiro et al., and Militaru C. et al., imply that resveratrol corrected the lipid profile, inflammatory status and quality of life of patients undergoing primary prevention of CVD [18,19,20]. It can be connected with its influence in many potential pathways. 

Inflammation associated with atherosclerosis is to a large extent regulated by the NF-κB pathway. It is logical to postulate that agents inhibiting or triggering the activation of this factor may play a significant role in atherogenesis [21]. The NF-κB itself is connected to various signalling agents by which can be activated and subsequently provoke inflammatory cascade. Studies in animals implicate that SIRT-1 is a potential target to focus on during the search for new solutions against atherosclerosis. The process of SIRT-1 upregulation may have a substantial impact on the activation of endothelium and its homeostasis [22,23]. SIRT1 is highly expressed in endothelial cells where it exercises control of angiogenesis through a wide variety of transcription regulators.

Resveratrol seems to be promising in its action limiting the inflammatory response at various levels. Experimental studies proved that resveratrol usage elevates the serum concentration of SIRT1 [24]. Pre-treatment of human vascular smooth muscle cells (VSMCs) at a dose 3–100 µM considerably enhanced SIRT1 expression [25]. Kao et al. [26] also noticed an augmentation of SIRT1 mRNA in human umbilical vein endothelial cells after pre-treatment with various doses of resveratrol (10–100 µM). Mechanism of sirtuin’s influence at molecular level have been linked to the prevention of atherosclerosis in many proposed models. It is postulated that sirtuin-1 moderates transcription factor RelA/p65 at K310 by deacetylation. What follows is suppression of its binding to naked DNA in human aortic endothelial cells. The changes eventually interfere with NF-κB signalling pathway activation, thereby restraining the expression of genes coding cell adhesion molecules: VCAM-1 and ICAM-1 [26,27]. What is more, SIRT-1-related suppression of NF-κB signalling pathway results in inhibition of synthesis of a number of pro-inflammatory cytokine, including: TNF-α, IL-1β, IL-6 and MCP-1 [28]. Interestingly, SIRT-1 upregulation is also able to lower angiotensin II type I receptor expression in VSMCs. Such changes may cause limitation in vessel contractility contributing to the prevention against hypertension and thereby anti-sclerotic effect [29]. Thus, an increase in SIRT-1 activity has been connected with a decrease in atherosclerotic lesion size and macrophage content in aortic arches [28]. Furthermore, SIRT1 transgenic apolipoprotein E null (apoE–/–) mice had fewer atherosclerotic lesions [30]. Zhang QJ et al., suggested that SIRT-1 overexpression may impede atherogenesis by influencing endothelial function through the alterations involving nitric oxide synthase (NOS-3) [31]. The explicit mechanism of SIRT1 activation by resveratrol remains unspecified, however it is considered that abovementioned polyphenol activates SIRT1 indirectly [31,32]. One of the potential mechanisms is the induction of AMPK (5′ adenosine monophosphate-activated protein kinase). This kinase affects the intracellular AMP-to-ATP concentration ratio, which indirectly increases the level of nicotinamide adenine dinucleotide (NAD+). Increased concentrations of NAD+ are able to enhance SIRT1 activity, considering that NAD+ is substrate for the enzyme [33].

Low levels of adiponectin in serum have been associated with weight gain and visceral fat increase. A noticeable reduction of adiponectin serum concentrations in obese and insulin-resistant states has been observed [34]. Observational human studies imply that a decrease in adiponectin levels may contribute to a development of cardiometabolic disorders [35]. This conclusion may result from certain evidence presenting adiponectin deficit as a risk factor of atherosclerosis [36]. Studies in animals ascribe to adiponectin an ability to restrain formation of atherosclerotic lesions [21,37]. Induction of adiponectin expression by resveratrol was described in animal studies, nevertheless some of the results remain contradicted. On the one hand, month long 10 mg/kg resveratrol pre-treatment of Wistar rats significantly heightened the level of adiponectin in blood serum [24]. Similar results have been obtained by Rivera et al. [38] in obese Zucker rats. They achieved an increase in the adiponectin serum concentrations after 8 weeks of 10 mg/kg resveratrol daily pre-treatment. This escalation was not observed in lean heterozygous littermates [39]. Following studies exploiting experimental rat models proved that 6 weeks of both high-dose resveratrol administration (200 mg/kg daily) and smaller dosage (6 weeks of 15 mg/kg) [40] are able to elevate the adiponectin concentration and its release from adipose tissue (Table 1). On the other hand, Palsamy and Subramanian [41] have not received a significant change of plasma adiponectin levels after 30 days of low-dose resveratrol treatment (5 mg/kg) in a healthy population of Wistar rats, although the raise were noticeable in a diabetic subjects [41]. The exact molecular mechanisms of adiponectin beneficial actions are not fully clarified but it can be assumed that moderating inflammatory response serves a crucial role once again. Studies show that adiponectin suppresses the nuclear translocation of NF-κB lowering the endothelial synthesis of pro-inflammatory chemokine IL-8 [42], TNF-α-induced expression of adhesion molecules on vascular endothelial cells and prevents monocyte adhesion which constitutes the initial step of atherogenesis [35].

Abovementioned aspects contributing to the limitation of inflammatory response by resveratrol may be linked to each other at the transcriptional level. RV is considered to upregulate SIRT1, FoxO1 and adiponectin transcription via interconnecting gene modulation pathways [52]. What is more, adiponectin may be correlated with a SIRT1-independent mechanism acting by induction of the AMPK, or as a FoxO1 activator through phosphoinositide-dependent kinase 1/protein kinase B signalling downregulation. Additionally, resveratrol effects on adiponectin indirectly by altering level of disulphide bond-A oxidoreductase-like protein [53].

## 3. Anti-Platelets Effect 

One of the major causes of cardiovascular diseases such as myocardial infarction, stroke or acute limb ischemia is a thromboembolic event provoked by excessive or abnormal platelet aggregation. Antiplatelet drugs are widely used in the prevention of the above-mentioned diseases [54]. Research conducted on resveratrol suggest its antiplatelet properties both in vitro [5,55] as well as in vivo. It seems that the mechanism of resveratrol activity on platelets is to a large extent focused on the stronger inhibition of COX-1 in relation to COX-2 [56]. Selective inhibition of COX-1 results in reduced synthesis of TxA2 (thromboxane A2), which is a potent triggering factor of platelet aggregation [57]. COX-2, per contra, occurring inter alia in vascular endothelial cells, synthesizes prostacyclin, which is an antiplatelet aggregator [6,58]. In this case, selective COX-1 inhibition appears to be the reason for the antiplatelet action. Interestingly, in Dutra et al.,’s study from 2017 [59] concerning derivatives of resveratrol, researchers created a resveratrol-furoxan hybrid compound able to release NO (nitric oxide) and inhibit platelet aggregation in the ADP agonist, collagen and arachidonic agonist pathway. Administration of this compound was connected with reduced bleeding time compared to acetylsalicylic acid (ASA) and protected up to 80% against thrombotic events in vivo (performed on mice). The above study shows the meaningfulness of further research and efforts to synthesize new resveratrol derivatives with much better properties.

## 4. Vascular Reactivity 

Vascular contractility is a significant factor in atherogenesis, as it is considered clinically relevant that arterial hypertension aggravates atherosclerosis [60]. Peripheral vascular resistance serves an influential role in pathogenesis of primary hypertension (also called essential or idiopathic). Arteries in patients suffering from hypertension often present augmented reactivity to contractive stimulus in comparison to healthy individuals. The exact cause of the phenomenon, however, remains unclear [61,62]. Due to hypertension, oxidative stress in the vascular wall increases which contributes to changes in metabolism and induces endothelium dysfunction, cell migration and proliferation of VSMCs [60]. Furthermore, the level of acute-phase proteins circulating in the bloodstream increases, which have been proven to activate the inflammation process through TLR-4 signalling pathways [61]. In various studies vascular contractile reactivity was evaluated and the mechanisms responsible for the reduction of the aforementioned atherogenic factors were assessed. It has been revealed that resveratrol may inhibit Ca2+/calmodulin cyclic nucleotide PDE (phosphodiesterase) and contribute to diminishment of VSMCs contractile response in partially PDE1 dependent manner.

Research conducted in rat models suggest that hypertension may be correlated with the increase of PDE1 expression and activation [63]. It has been stated that inhibition of PDE1 leads to decrease of arterial contractile response as consequence of intracellular cGMP concentration increase [64]. The subtype 1C of PDE is expressed in proliferating smooth muscle cells and may be potentially involved in atherogenesis [65]. If the inhibition of PDE1C by resveratrol is presented to be relevant in treatment, one additional advantageous effects would be a slowdown of VSMCs proliferation which remains the one of the fundamental elements of atherosclerotic plaque development [66]. Park et al., described resveratrol [67] to be a potent antagonist of cAMP PDEs (including PDE1-4) that inhibits these enzymes directly in a concentration-dependent manner. Kline and Karpinski [68] observed resveratrol’s ability to induce NOS-3 in direct and indirect manners through AMPK, SIRT1 and nuclear factor erythroid 2-related factor 2 pathways. Additionally, they noticed that resveratrol acts directly on VSMCs by blocking the l-type calcium channel resulting in limitation of intracellular Ca2+ release.

## 5. Resveratrol Influence on Diabetes

There exists a close connection between DM (diabetes mellitus) and CVDs, which are the most common causes of morbidity and mortality in diabetic patients. Type 2 diabetes is a condition where persistent hyperglycaemia and hyperinsulinemia are associated with chronic low-grade inflammation. As the consequence, the amount of ROS increases [69] which can have an impact on cell damage. Affected can be also neurons [70]. Bhatt et al. [43] in their studies in a group of 62 patients with type II diabetes compared the use of standard antidiabetic therapy with a combination of this therapy and resveratrol. After three months of treatment, the results in both groups were evaluated. The combination had a statistically significant advantage in positive effect. It caused a decrease in HbA1c (glycated haemoglobin A1c), lowered the systolic blood pressure, as well as total cholesterol level. It did not, however, have a statistically significant effect on body weight and respective lipoprotein fractions. Thirunavukkarasu et al. [45] achieved a reduction in glycaemia in the group of rats with DM2 receiving resveratrol. On the other hand, in a randomized, double-blind study of Bo et al. [71] conducted on a group of 192 people suffering from DM2, the use of resveratrol did not bring any statistically significant changes in biochemical markers such as: CRP (C-reactive-protein), BMI (Body Mass Index), blood pressure, HbA1c and others. In the work of Öztürk et al. [72] which has collected a dozen clinical trials investigating the effect of resveratrol on DM2, researchers have noticed the pleiotropic effects of resveratrol. In attempt to describe potential mechanisms of its profitable actions a broad number of factors have to be considered.

One of the possible mechanisms once more focused on the activation of abovementioned SIRT-1 [73]. Studies have shown a significant reduction in its expression and activity both in vitro and in vivo in the course of DM2 [74,75]. Some of the positive effects of resveratrol may be explained by activation of AMPK. Mentioned kinase regulates intracellular processes such as energy metabolism, mitochondrial functions and cellular homeostasis. AMPK dysregulation correlated with insulin resistance and hyperglycaemia-associated tissue damage suggesting the role of AMPK in DM2 [72,76]. Furthermore, it is hypothesized that the beneficial effect in diabetes can also be explained by the activation by the SIRT-1 of the PGC-1α cascade (peroxisome proliferator-activated receptor gamma coactivator 1-alpha) [76]. PGC-1α, as a transcriptional coactivator, regulates genes involved in energy metabolism. It is one of the main regulators of mitochondrial biogenesis [77]. Mootha et al. [78] in their studies described a reduced level of transcription of the PPARGC1 gene (gene encoding PGC-1α) in calf muscles of diabetic patients. What is more, impaired mitochondrial function (associated with less PGC-1α activity) promoting fatty acid accumulation, as opposed to oxidation, can significantly contribute to intracellular lipid accumulation, which is associated with insulin resistance in DM2 in humans [79]. Based on resveratrol PGC-1α cascade activating abilities, some positive influence od RV may be assumed. 

In DM2, pancreatic β-cell damage is related to increased creation of free radicals [80,81]. One possible mechanism of resveratrol usage in DM2 may be its antioxidant effect. In the studies of Brasnyó et al. [44] a decrease in insulin resistance in patients receiving resveratrol has been shown. Researchers linked it to increased activation of the Akt signalling pathway. In addition to direct antioxidative activity, it is suggested that resveratrol may affect the expression of genes regulating pro and antioxidant mechanisms by reducing the expression of enzymes responsible for the production of free radicals and increasing the production of those involved in scavenging of ROS as NADPH oxidase (Nox) and its products: SOD (superoxide dismutase) and GPx1 (glutathione peroxidase 1) [82].

Another potentially advantageous action of resveratrol in DM2 is an attenuation of the NF-κB signalling pathway [83,84,85]. NF-κB is a protein complex that regulates the immune response and can be considered as prototypical proinflammatory factor in many diseases [86]. Researchers [87] propose a model in which activation of NF-κB results in increased production of IL-6, which induces insulin resistance in hepatocytes [88,89]. In this case, resveratrol reducing the activation of this pathway could affect the decrease of insulin resistance in the tissues. DM2 is often associated with abdominal obesity [90], which can lead to metabolic syndrome, abdominal adiposity and hepatic steatosis (fatty liver). All the states result in persistent low-grade inflammation being a cause of oxidative stress [89]. Cai et al. [89] in their study found that the NF-κB pathway is activated in rodent livers by two obesity models: HFD (High Fat Diet) and genetic hyperphagia.

Chronic hyperglycaemia generates AGEs (advanced glycation end products) and their RAGE receptors [91]. RAGEs activation is another trigger factor of NF-κB transcription cascade [92]. This suggests that activation of NF-κB in diabetic patients correlates with the quality of glycaemic control [93]. The reduction of NF-κB activity by resveratrol in numerous ways provides a potential protection line against lasting hyperglycaemia. Interdependence of described numerous mechanisms is evident, what brings both many opportunities and obstacles.

## 6. Cerebral Blood Flow

Chronic systemic diseases are thought to impair vasorelaxation with the consequence that cerebral blood flow is diminished [94]. Cognitive impairment and dementia are characterized by defective cerebrovascular blood flow which is considered to be a significant element in their pathogenesis. Moreover, Araya et al., state that cerebrovascular abnormalities, especially in cerebral microvessels, potentially lead to neuronal dysfunction and cognitive impairment [94,95]. Maintenance of cerebral blood flow at both stable and sufficient levels seems to be a potential target in the pharmacological prevention of neurodegeneration. Beneficial effect of resveratrol treatment has been shown in disorders such as Alzheimer’s disease, Parkinson’s disease, Huntington’s disease, amyotrophic lateral sclerosis [96] and vascular dementia [97].

Resveratrol increases BDNF serum concentrations which, according to literature, reflects an increase of BDNF in brain parenchyma. Potentially, the aforementioned neurotrophin constitutes the link in maintaining cerebral blood flow in response to hypoxic stress. Guo et al., suggest that BDNF seems to be serving a major role in the neurovascular unit of brain. Their results confirm that cerebrovascular endothelium can secrete potent neuroprotective agents [98]. BDNF is involved in the differentiation and maturation of nerve cells in the central nervous system. The neurotrophin is also associated with increased ratio of growth, formation of new neuronal connections and nerve branching, as well as induction of synaptic transmission [99,100,101]. The diminishment of serum BDNF levels may result in aggravation and poor outcome in neurodegenerative diseases [102,103,104,105]. Accordingly, agents like resveratrol that induce the expression of BDNF are believed to reproduce the biological effects of the neurotrophin.

Induction of BDNF expression in brain structures following an administration of naturally existing plant-derived polyphenols was previously described by Jeon et al. [106]. Zhang et al., found that resveratrol induces BDNF release from astroglia in rat primary astroglia-enriched cultures suggesting that resveratrol administration may be more efficient than direct treatment with neurotrophic factors [107]. The mechanism of BDNF upregulation by resveratrol has not been explained comprehensively yet. According to Goggi et al., the release of BDNF depends on the concentrations of both extracellular and intracellular calcium. They have also noticed that BDNF release is link to the activation of IP3 (inositol trisphosphate) mediated Ca^2+^ release from intracellular stores. BDNF was also modulated by receptors coupled to adenylate cyclase. Another probable mechanism is activation of the CREB and ERK1/2 signalling pathways which result in an increased production of neurotropic factors [107]. 

Resveratrol has the ability to induce NOS-3 in both a direct and indirect manner through AMPK, SIRT1 and Nrf2 pathways and, as a result, it positively affects vasorelaxation in cerebral arteries [108]. Results presented in study of Leblais et al., state that resveratrol may directly act on VSMCs promoting pulmonary artery relaxation via different mechanisms including induction of guanylyl cyclase, inhibition of protein kinase C, activation of smooth muscle K+ channels, or acting via Ca2+ [109]. Direct reduction of VSMC contractility by resveratrol may be a meaningful mechanism in neuroprotection since pathogenesis of neurodegenerative diseases is also matched with vasoconstriction [110]

One of the most prevalent CV illness is stroke [111]. During ischemia, the increased production of free radicals by mitochondria becomes responsible for endothelial dysfunction and causes excitation contraction coupling impairment in VSMCs [112]. The direct cell damage resulting from ischemia leads to death, apoptosis or metabolic changes. Insults caused by stroke must be distinguished between primary and secondary. The former cause unavoidable damage in the centre of the ischemic area. Secondary ones result from processes lasting days in the tissues surrounding the primary injury. Induced oedema, release of lethal calcium ions amounts, epigenetic changes and agents created by activated microglia are directly or indirectly toxic to neurons and initiate progressive damage [113,114]. In experimental studies on the mouse model, WenPeng Dong and co-workers assessed the effect of resveratrol on the extent of damage caused by ischemia and reperfusion. [46] (Table 1). The area of ischemia and microcirculatory injuries were significantly smaller compared to the control group not receiving resveratrol. Similar results were obtained by Huang et al., and Sinha K. et al., where resveratrol managed to reduce infarct volume and prevented impairments in motor function in rats. [47,48,49].

Although mechanisms underlying the beneficial effects are yet still to be elucidated, there exist a supposition that angiogenesis mediated by VEGF and MMP-2 might be responsible for insult limitation [50]. Ischemic cerebral regions showed significantly higher concentrations of abovementioned proteins. [114]. What is more, the alteration of mitochondrial function via SIRT-1 target mitochondrial uncoupling protein 2 (UCP2) caused by RV may be a way to mimic ischemic preconditioning [115]. UCP2–/– mice were described to be less vulnerable to microglia activation and consequent unfavourable effect [116]. Since SIRT 1 inhibitor tended to prevent UCP2 upregulation, the hypothesis of sirtuin involvement in the neuroprotection seems reasonable [115]. Anti-inflammatory effects where presented in work of Wang Q et al., in which RV diminished neuronal cell death and glial activation in the hippocampus of gerbils after artificially induced common carotid artery occlusion [51]. 

Above all, the most anticipated still remains a perspective of therapeutical application in human. Long-term observation of the influence of the administration of resveratrol on secondary prevention of stroke confirmed its beneficial effects (both in the 100 mg dose and 200 mg/day) on a number of risk factors for recurrence [116]. There was a significant improvement in glucose profile, lipidogram and arterial pressure. During the 12 months of the study, Katalin Fodor et al., they did not detect a single vascular incident [117]. 

## 7. Conclusions

The information presented above allows for considering resveratrol as a promising drug in the treatment of cardiovascular conditions. The moderation of free radicals creation and proinflammatory response diminishment may prove to be helpful in slowing down atherosclerosis development as well as in limiting the changes connected to chronic hyperglycaemia. Potential properties stimulating neuronal renewal, if proven, would find application in the treatment of various forms of dementia. If resveratrol is demonstrated to have clinically meaningful anti-sclerotic activity in humans, one potential application may be to reduce the burden of certain neurodegenerative disorders. In perspective of future findings, it is worth to consider the use of not only resveratrol alone but also its derivatives with preferable effects. Studies assessing beneficial effects of RV on cardiovascular system need to be strengthened in order to plausibly evaluate its usability. Wide spread of dosage used with similar effect makes it difficult to determine the proper dose. Additional studies are essential to verify efficacy of resveratrol in conditions specified in the paper. 

## Figures and Tables

**Table 1:** Oral administration of resveratrol in vivo trials.

Authors	Subject of Study	Dose	Result
**Tomé Carneiro et al. [19]**	Human with coronary artery disease	Polyphenolic composition + 8.1 ± 0.5 mg resveratrol per capsule. 1 capsule/day in the morning for the first 6 months and 2 capsules/day for the following 6 months	↑ serum adiponectin ↓ (PAI-1)
**Militaru C. et al. [20]**	Human with stable angina pectoris	20 mg/day of resveratrol	↓ hs-CRP, ↓ NT-proBNP, ↓ total cholesterol, ↑ quality of life
**Bhatt et al. [43]**	Human with DM2	250 mg/day of resveratrol	↓ HbA1c, ↓ SBP, ↓ total cholesterol
**Brasnyó et al. [44]**	Human with DM2	2 × 5mg/day of resveratrol	↓ insulin resistance, ↑ pAkt: Akt
**Wiciński et al. [2]**	Wistar rats	10 mg/kg of resveratrol per day	↑ serum BDNF
**Wiciński et al. [24]**	Wistar rats	10 mg/kg of resveratrol per day	↑ serum adiponectin
**Rivera et al. [38]**	Zucker rats	10 mg/kg of resveratrol per day	↑ serum adiponectin
**Beaudoin et al. [39]**	Zucker rats	200 mg/kg of resveratrol per day	↑ serum adiponectin and its release
**Thirunavukkarasu et al. [45]**	streptozotocin induced diabetic rats	2.5 mg/kg of resveratrol per day	↓ glucose level
**Dong et al. [46]**	Balb/c mice	50 mg/kg of resveratrol per day	↓ infract size after stroke, recover of neurologic function
**Huang et al. [47]**	Long-Evans rats	10^−6^–10^−9^ g/kg of resveratrol intravenous	↓ infract size after stroke
**Sinha et al. [48]**	Wistar rats	20 mg/kg of resveratrol intraperitoneal	prevents motor impairment, ↑ MDA, ↓ glutathione, ↓ infract size after stroke
**Fukuda et al. [49]**	Rats	10 mg/kg of resveratrol per day	↑ VEGF, ↑ Flk-1,3, ↑ NOS
**Della-Morte et al. [50]**	Rats	10–100 mg/kg of resveratrol intraperitoneal	↑ SIRT-1, ↓ UCP2
**Wang et al. [51]**	Mongolian gerbils	30 mg/kg of resveratrol intraperitoneal	↓ DND, ↓ glial activation

Polyphenolic composition is (~25 mg anthocyanins, ~1 mg flavonols, ~40 mg procyanidins and ~0.8 mg hydroxycinnamic acids), ↓—reduction, ↑—increase, PAI-1—Plasminogen activator inhibitor-1, hs-CRP—high-sensitivity C Reactive Protein, NTproBNP—n-terminal prohormone of brain natriuretic peptide, quality of life—measured in the number of angina pectoris episodes and the amount of nitroglycerin used, HbA1c—Glycated haemoglobin A1c, SBP—systolic blood pressure, BDNF—brain-derived neurotrophic factor, MDA—Malondialdehyde, VEGF—vascular endothelial growth factor, Flk-1,3—tyrosine kinase receptor of VEGF , NOS—nitric-oxide synthase, SIRT1—sirtuin 1, DND—delayed neuronal cell death, UCP2—mitochondrial uncoupling protein 2.
